# Occupational and Environmental Determinants of Musculoskeletal Disorders Among Nurses in Conflict-Affected Gaza Hospitals

**DOI:** 10.5334/aogh.5055

**Published:** 2026-01-20

**Authors:** Ahmed Z. Eleiwa, Khalid Jamal Khadoura

**Affiliations:** 1 Faculty of Nursing, The Islamic University of Gaza, Gaza, Palestine; 2 Faculty of Medical Sciences, Department of Nursing, Israa University, Gaza, Palestine

**Keywords:** musculoskeletal disorders, occupational health, nurses, ergonomic risk factors, conflict settings, Gaza Strip

## Abstract

*Background:* Musculoskeletal disorders (MSDs) are a major occupational health concern among nurses, particularly in high-stress and resource-constrained armed conflict settings. This study assessed the prevalence and occupational and environmental correlates of MSDs among nurses caring for critically ill patients in Gaza City hospitals during the 2023–2025 conflict.

*Methods:* A cross-sectional descriptive analytic study was conducted among 172 nurses in intensive care units (ICUs), emergency departments, operating rooms, and pediatric ICUs. Data were collected using a structured questionnaire adapted from the Arabic Nordic Musculoskeletal Questionnaire and ergonomic exposure assessment tools. Descriptive statistics summarized MSD prevalence and workplace exposures. MSD burden, defined as the number of affected body regions over the past 12 months, was analyzed using negative binomial regression, with logistic and multiple linear regression used as exploratory sensitivity analyses.

*Results:* The 12-month prevalence of MSDs was high, most commonly affecting the neck (69.2%), lower back (68.0%), and shoulders (64.5%). Pain intensity was moderate to high, particularly in the lower back (mean 5.31 ± 3.08), and over 70% of nurses reported difficulty performing work tasks due to pain. Ergonomic risks were widespread, including prolonged neck flexion (84.3%) and trunk bending (67.4%). In multivariable negative binomial regression, being married was independently associated with fewer affected body regions (adjusted incidence rate ratio [aIRR] = 0.71, p = 0.002), while working in humid environments was associated with higher MSD counts (aIRR = 1.24, p = 0.026). Adequate rest showed a modest protective association (aIRR = 0.82, p = 0.045). Work pace and perceived stress were not independently associated with MSD burden.

*Conclusion:* Critical care nurses in conflict-affected Gaza experience a high burden of MSDs, particularly in the neck and lower back. Workplace humidity and insufficient rest were key modifiable correlates. Occupational health interventions are urgently needed to protect nurses and sustain care delivery.

## Introduction

Musculoskeletal disorders (MSDs) are a major occupational health concern among nurses worldwide, contributing to chronic pain, functional limitations, reduced work performance, and substantial healthcare costs. Nurses are particularly vulnerable due to the physical demands of patient handling, prolonged standing, repetitive movements, and sustained awkward postures. Recent systematic reviews and meta-analyses consistently demonstrate a high global prevalence of MSDs among nursing professionals [1].

A comprehensive systematic review and meta-analysis by Gorce and Jacquier-Bret reported an overall work-related MSD prevalence of 87.8% among nurses in Europe, with the lower back (61.4%), neck (49.9%), and upper back (46.3%) being the most affected anatomical regions. Similarly, a scoping review from sub-Saharan Africa identified a substantial burden of MSDs among nurses, particularly low back pain, highlighting the multifactorial etiology of these conditions, including physical workload, environmental exposures, and psychosocial stressors [2].

Unlike fast-paced but stable healthcare environments, conflict settings impose sustained infrastructural, environmental, and psychosocial stressors that fundamentally alter nurses’ exposure profiles and risk pathways.

Globally, MSD risk among nurses is shaped by an interplay of personal, occupational, and environmental factors. Individual characteristics such as age, sex, and body mass index interact with occupational exposures, including shift work, extended working hours, insufficient recovery time, and high work pace. Environmental and ergonomic hazards—such as inadequate equipment, poor workstation design, and adverse thermal conditions—further exacerbate musculoskeletal strain [3, 4].

However, in conflict-affected settings, these established risk factors may be substantially intensified. Healthcare workers operating in war zones face disrupted hospital infrastructure, shortages of essential equipment, overcrowded facilities, staff shortages, unpredictable workloads, and prolonged duty hours. In addition, exposure to repeated mass-casualty events, displacement, insecurity, and chronic psychological stress may indirectly increase musculoskeletal vulnerability by amplifying physical workload, limiting rest, and impairing recovery. Environmental conditions, such as poor ventilation, damaged buildings, and elevated humidity due to compromised infrastructure, may further contribute to ergonomic strain and discomfort.

Despite the growing literature on MSDs among nurses, empirical evidence from active conflict settings remains scarce. In particular, little is known about how occupational, environmental, and psychosocial stressors specific to armed conflict interact to influence MSD patterns and severity. Addressing this gap is essential for informing occupational health interventions tailored to crisis settings.

Therefore, this study aimed to assess the prevalence, anatomical distribution, and associated occupational and environmental factors of MSDs among nurses working in critical care units of Gaza City hospitals during the 2023–2025 conflict, with a specific focus on determinants shaped or intensified by prolonged armed conflict.

## Methods

### Study design and setting

A cross-sectional descriptive-analytic study was conducted to assess the prevalence and correlates of MSDs among nurses working in Gaza City hospitals during the 2023–2025 armed conflict. Data were collected from five major hospitals that remained operational under wartime conditions: Al-Shifa Medical Complex, Patient Friend’s Benevolent Society Hospital, Al-Ahli Arab Hospital, Public Aid Hospital, and Al-Helou International Hospital. These institutions were among the few operational facilities providing essential healthcare services and experienced substantial patient surges, infrastructure constraints, and workforce shortages during the study period under extreme wartime conditions.

### Participants

Registered nurses providing direct patient care in high-intensity departments (ICU, ER, OR, and PICU) were eligible. Due to movement restrictions, security risks, and staffing instability during the conflict, convenience sampling was employed as a pragmatic and ethically feasible approach. Nurses were included if they had worked continuously for at least three months during the conflict period. A total of 172 nurses participated. The use of convenience sampling was necessitated by the restrictive and volatile conditions of the ongoing conflict, including security constraints, staff shortages, and fluctuating hospital operations. Probability-based sampling or larger-scale recruitment was not feasible under these circumstances. While this approach limits generalizability, it reflects a pragmatic strategy commonly employed in research conducted in active conflict settings.

### Data collection

Data were collected using a structured, self-administered questionnaire comprising four sections:

1. **Demographic and occupational characteristics** (age, sex, marital status, BMI, experience, displacement history).
2. **Musculoskeletal symptoms,** assessed using the Arabic version of the Nordic Musculoskeletal Questionnaire (NMQ), previously validated and widely used in Arabic-speaking populations to assess 12-month and 7-day MSD prevalence and pain intensity.
3. **Ergonomic and postural exposures,** adapted from standardized workplace posture assessment tools (including REBA-informed items).
4. **Psychosocial and environmental factors,** including work pace, perceived stress, rest adequacy, and environmental conditions (humidity, temperature, workspace adequacy), adapted from the Karasek Job Content framework.

The questionnaire was reviewed by a panel of occupational health and nursing experts to ensure contextual relevance to conflict settings.

### Pilot testing

A pilot study involving approximately 10% of the sample was conducted to assess clarity, feasibility, and completion time under wartime conditions. Minor wording adjustments were made to improve comprehension; no structural changes were required. Pilot data were not included in the final analysis.

### Data analysis

Data were analyzed using STATA version 14. Descriptive statistics summarized participant characteristics, MSD prevalence, pain intensity, ergonomic exposures, and environmental conditions. Given the modest sample size, inferential analyses were considered exploratory and interpreted with caution, particularly regarding effect estimates and statistical precision.

MSD burden was defined as the number of affected body regions over the preceding 12 months. Because this count outcome exhibited overdispersion, negative binomial regression was used for univariable and multivariable analyses. Variables with theoretical relevance or p < 0.20 in univariable analyses were included in multivariable models. Multicollinearity among predictors was assessed using variance inflation factors (VIF) in a multiple linear regression model, and all VIFs were below 5, indicating acceptable collinearity.

As sensitivity analyses, logistic regression (presence of any MSD) and multiple linear regression were also conducted to explore the robustness and direction of associations across alternative outcome specifications. Overall, the negative binomial regression model provided the best fit to explain the data. All analyses were interpreted cautiously, recognizing the exploratory nature of inferential findings given the sample size and study design. Statistical significance was set at p < 0.05.

### Ethical considerations

The study protocol was approved by the Islamic University of Gaza Research Ethics Committee (approval number: IUG/1086/2025). Additional administrative permissions were obtained from the Ministry of Health (approval number: MOH/25118162/8/2025) and participating hospitals. Written informed consent was obtained from all participants, ensuring voluntary participation, confidentiality, and anonymity. Participants were informed of their right to withdraw at any point without penalty, and all data were used exclusively for research purposes.

## Results

### Participant characteristics

A total of 172 nurses from five Gaza City hospitals participated. The majority worked at Al-Ahli Arab Hospital (43.0%), followed by Public Aid Hospital (25.6%), Al-Shifa Medical Complex (13.4%), Al-Helou International Hospital (10.5%), and Patient Friend’s Benevolent Society Hospital (7.6%). Most participants were male (59.3%), with a mean age of 33.1 ± 6.2 years (range: 22–56), and an average professional experience of 9.2 ± 5.8 years.

Most nurses held a bachelor’s degree (69.2%), were married (76.7%), and had a normal BMI (65.7%) (Table 1). Participants reported a mean of 6.9 ± 4.6 displacement events (range: 0–20) during the 2023–2025 war, the majority experienced 5–10 displacements.

**Table 1:** Demographic and professional characteristics of participants (N = 172).

VARIABLE	CATEGORY	N	%
Hospital	Al-Shifa Medical Complex	74	43.0
	Patient Friend’s Benevolent Society	44	25.6
	Al-Ahli Arab Hospital	23	13.4
	Public Aid Hospital	13	7.6
	Al-Helou International Hospital	18	10.5
Department	PICU	27	15.7
	OR	53	30.8
	ER	37	21.5
	Other	54	31.4
Job title	Practical nurse	31	18.0
	Registered nurse	122	70.9
	Supervisor	8	4.7
	Head nurse	10	5.8
Education	Diploma	32	18.6
	Bachelor	119	69.2
	Master or above	21	12.2
Gender	Male	102	59.3
	Female	70	40.7
Marital status	Married	132	76.7
	Single	36	20.9
	Divorced/Widowed	4	2.3
BMI category	Underweight	5	2.9
	Normal	113	65.7
	Overweight	44	25.6
	Obese	10	5.8
Years of experience	Mean ± SD	9.2 ± 5.8	1–27
Age (years)	Mean ± SD	33.1 ± 6.2	R(22–56)
Displacement during war	Mean ± SD	6.9 ± 4.6	R(0–20)

R: Range, SD: Standard deviation.

In our study, nearly 60% of participants were male, which diverges from the gender distribution typically seen in nursing workforces in many high-income settings where females predominate. This reflects the local workforce composition in Gaza, where nursing—including in critical care units—has a higher proportion of male clinicians, a pattern that is shaped by regional sociocultural norms and the demands of working in an active conflict environment. Although some non-conflict literature suggests gender differences in MSD prevalence, findings are inconsistent and often context-specific. In our cohort, gender was not significantly associated with MSD count in either univariable or multivariable models, indicating that occupational and environmental exposures common to all nurses—such as prolonged high work pace, awkward postures, and adverse environmental conditions—are likely the dominant drivers of MSD burden rather than gender alone.

### Prevalence of musculoskeletal disorders

**Figure 1:**
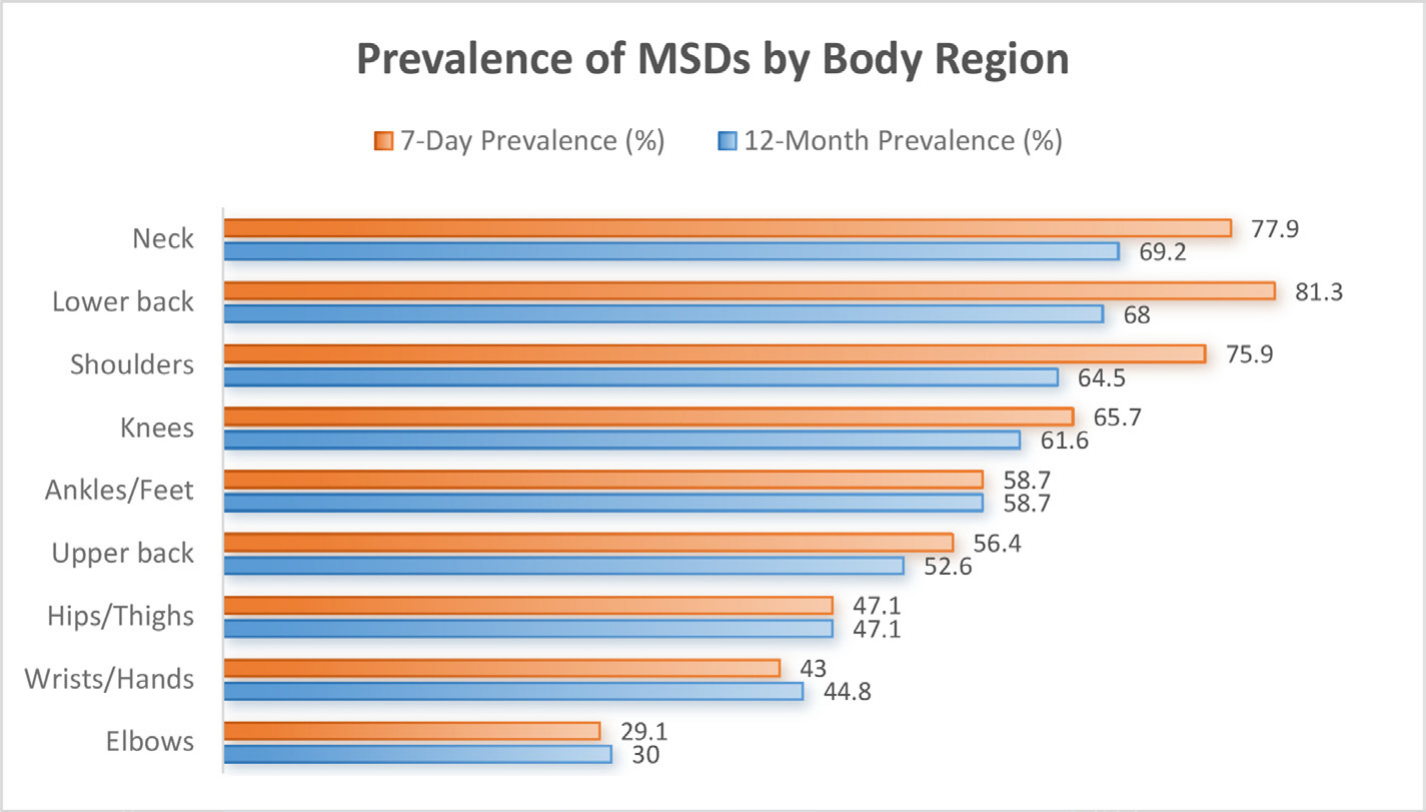
Prevalence of musculoskeletal symptoms by body region (N = 172).

The 12-month prevalence of musculoskeletal symptoms was highest in the neck (69.2%), lower back (68.0%), and shoulders (64.5%), followed by knees (61.6%) and ankles/feet (58.7%). The 7-day prevalence showed a similar pattern, with lower back (81.3%), neck (77.9%), and shoulders (75.9%) most affected (Figure 1).

### Pain intensity and work absence

**Figure 2:**
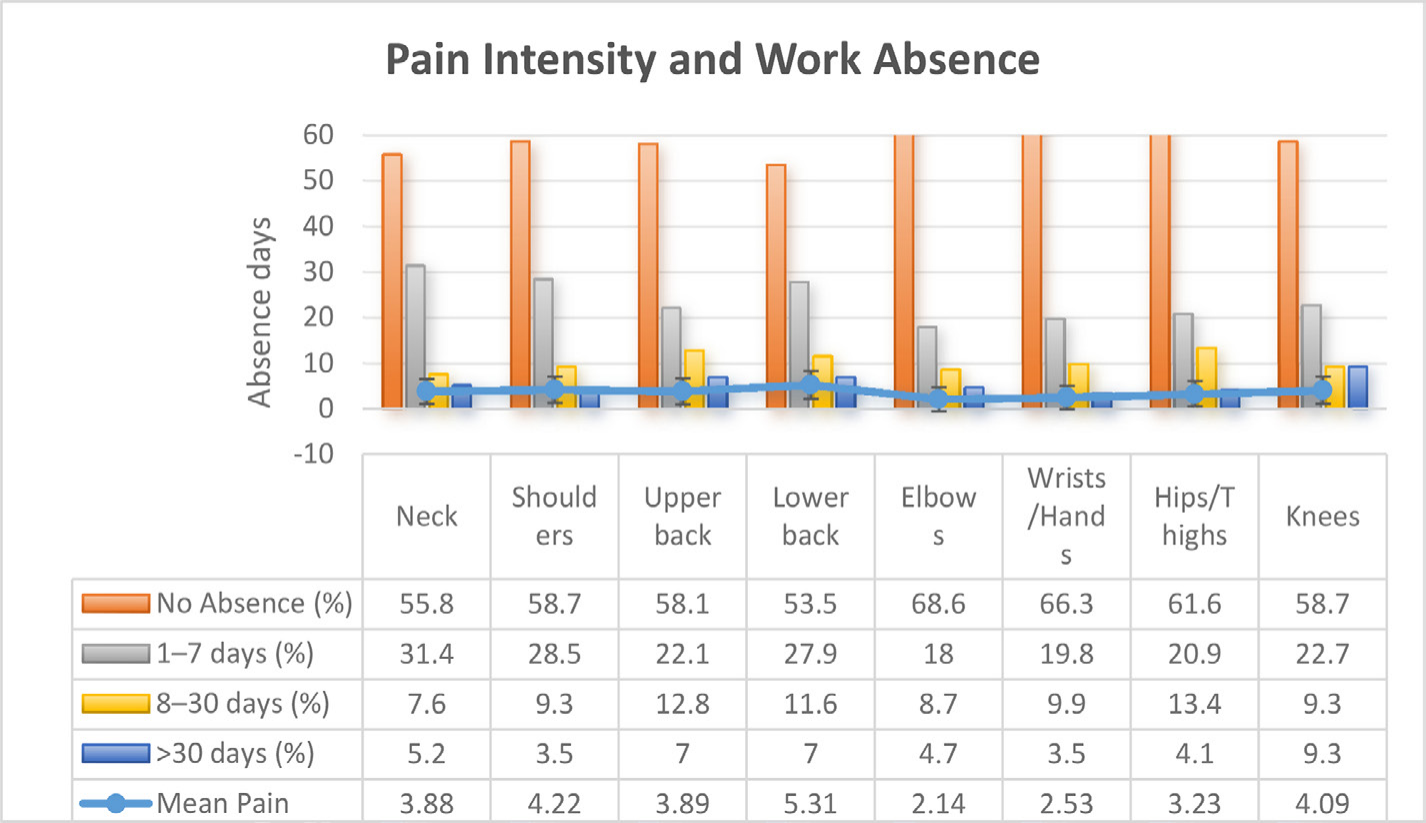
Mean pain intensity and work absence by body region (N = 172).

Mean pain intensity (0–10 scale) was highest in the lower back (5.31 ± 3.08), followed by shoulders (4.22 ± 2.86) and knees (4.09 ± 2.99). Short-term work absence (1–7 days) was most common, whereas prolonged absences (>30 days) were rare (Figure 2).

### Laterality of musculoskeletal pain

**Figure 3:**
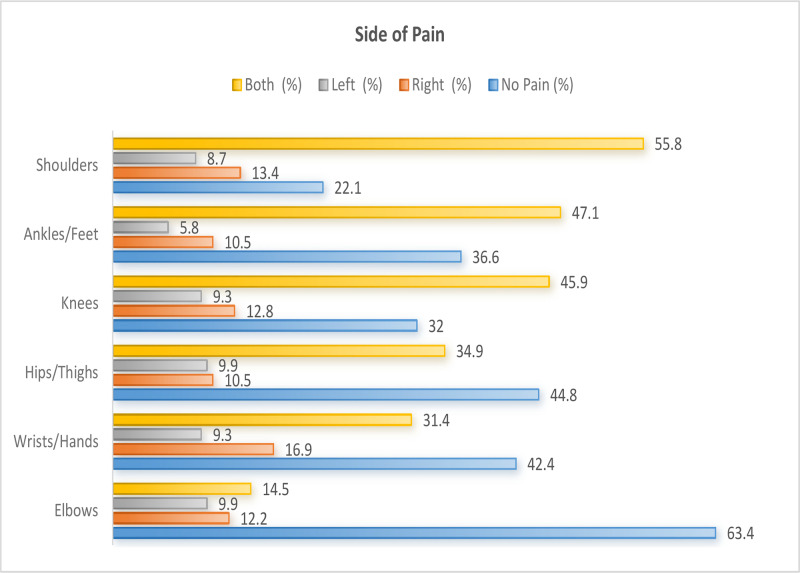
Distribution of musculoskeletal pain by body side (N = 172).

Bilateral pain was most frequent in the shoulders (55.8%), ankles/feet (47.1%), and knees (45.9%). Right-sided pain predominated in elbows (12.2%) and wrists (16.9%), whereas left-sided pain was less common (Figure 3).

### Work limitation and job/task changes

**Figure 4:**
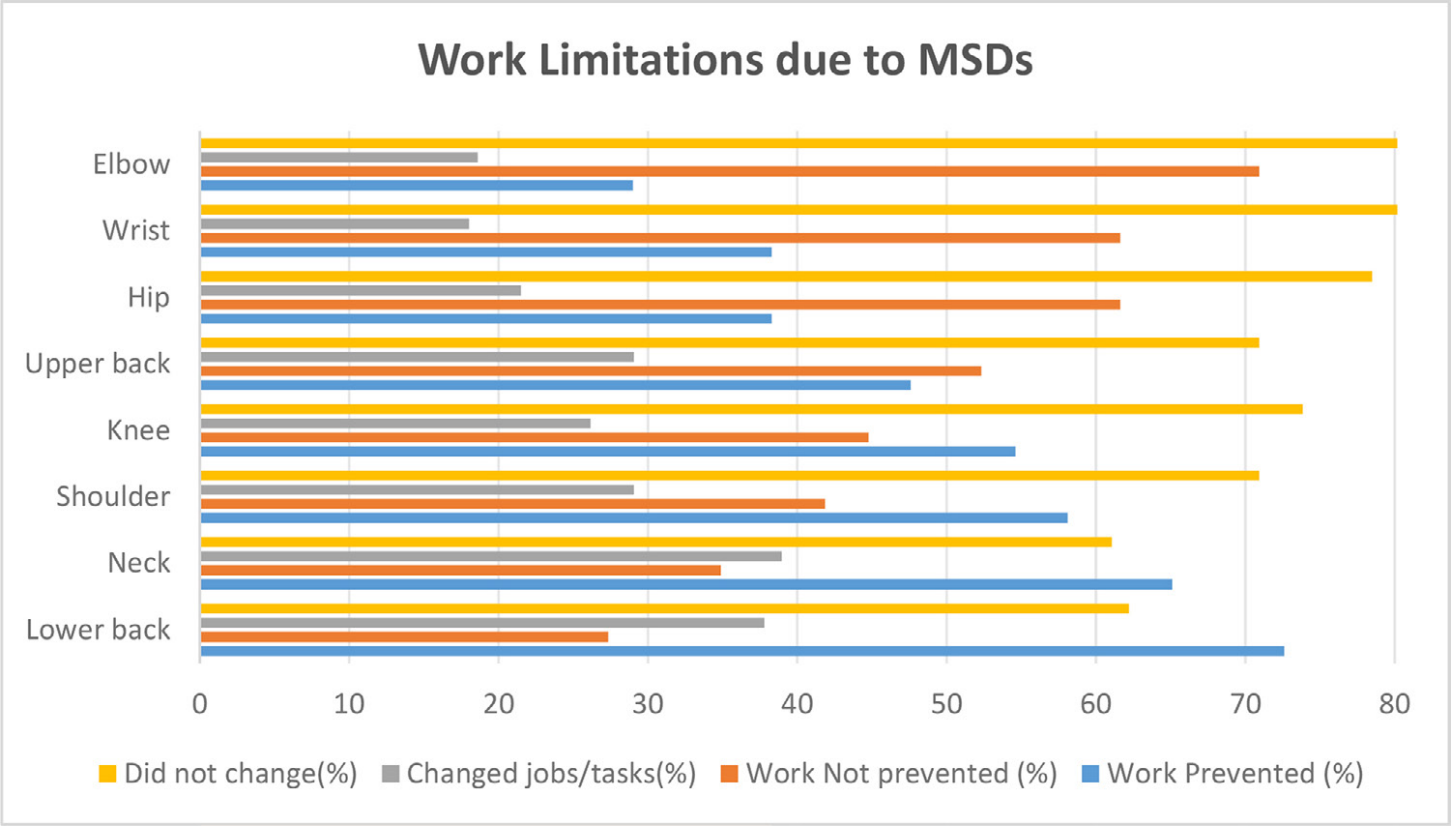
Work limitations reported by participants with MSDs. Limitations include absenteeism, reduced productivity, and changes in work tasks.

Lower back and neck symptoms were most frequently associated with work limitation (72.7% and 65.1%, respectively), whereas elbow and wrist involvement had lower impact (29–38%). Voluntary job or task changes due to chronic MSDs were less frequent, with neck (38.9%) and lower back (37.8%) being the most common. Changes due to accidents were rare (7–15%) (Figure 4).

### Working postures

Participants reported frequent exposure to awkward and sustained postures, including trunk and neck flexion, prolonged bending or twisting, repetitive wrist/arm movements, and static lower-limb postures, representing ergonomic risk factors for MSDs. Most workers adopt moderate to high trunk and neck flexion, with prolonged bending, twisting, and frequent turning being common. Upper limb activities, including repetitive wrist flexion/extension and arm twisting, are also highly prevalent. For the lower limbs, sitting is the most common posture, while prolonged standing and bent/twisted legs affect nearly half of participants. These findings highlight substantial ergonomic risks and suggest that interventions to optimize posture and reduce repetitive or sustained movements are warranted to prevent work-related MSDs, full details are available on supplementary file 1.

### Work environment factors

- Workload and scheduling characteristics
  The average working day among nurses was 11.19 ± 5.39 hours, with an average of 5.25 ± 1.33 working days per week. Nearly half of the participants (48.8%) reported working 6–8 hours per day, while 30.2% worked 9–12 hours, and about one in five (21%) reported extended shifts exceeding 12 hours per day. Such long and irregular working hours reflect the extraordinary operational demands faced by nurses in Gaza City hospitals during the 2023 war period, where staff shortages and patient surges required prolonged duty hours.
- Work environment conditions
  Table 3 shows the distribution of key environmental and ergonomic conditions reported by nurses. A large majority (86.1%) indicated that they often worked overtime, while only 33.1% reported having enough workspace and 30.2% had lumbar support on their seats.
  Approximately one-third (37.2%) had adjustable benches, and only about half (52.9%) said they could change their posture freely. Conversely, 62.8% reported maintaining the same posture for most of their shift, and 73.8% found their posture uncomfortable. Environmental discomfort was also notable: 36.1% reported feeling cold, 66.3% reported humidity, and 73.8% felt they lacked enough rest time. Only 26.2% indicated they could rest adequately, and 33.1% could rest regularly during working hours. Psychological stress and workload pressure were highly prevalent. About 79.1% of nurses felt stressed at work, and 76.2% reported difficulty keeping up with the work pace. These findings reflect intense psychosocial demands consistent with emergency and war-related healthcare contexts, full details are available on supplementary file 1.

The data indicate widespread ergonomic and psychosocial strain among nurses working during the 2023 Gaza conflict. Prolonged duty hours, lack of workspace and lumbar support, and static or awkward postures were common. Nearly three-quarters of the nurses experienced discomfort from posture and humidity, while rest opportunities were limited. Psychosocially, high levels of stress and pace pressure suggest that emotional exhaustion and cognitive overload were pervasive. These adverse work conditions likely exacerbated the prevalence of MSDs documented earlier, underscoring the urgent need for ergonomic adjustments, rest schedules, and stress-management interventions in hospital settings under crisis conditions.

### Association between participants characteristics and work-related factors with musculoskeletal disorders among nurses

The prevalence of MSDs in the past 12 months was high across all demographic groups. The association between participants characteristics and work-related factors with MSDs in the past 12 months was examined using chi-square tests for categorical variables and independent t-tests for continuous variables. Table 2 illustrates the categorical factors, no statistically significant associations were found between MSDs and education level (p = 0.884). Likewise, job title was not significantly associated with MSDs (p = 0.919), suggesting that all nursing roles were exposed to similar occupational stressors during wartime conditions. Also, no significant association was observed between gender and MSDs (p = 0.334). However, a significant relationship was found between marital status and MSDs (χ² = 6.2, p = 0.011), with married nurses compared to unmarried (single, divorces, and widowed) showing higher prevalence—possibly reflecting greater physical and emotional strain from balancing family and professional responsibilities. Although overweight and obese nurses reported more MSDs, the association between BMI category and MSDs did not reach statistical significance (p = 0.134). Furthermore, no statistically significant associations were found between MSDs and work-related factor, such as overtime, workspace type, lumbar support, adjustable benches, posture freedom, maintaining the same posture, uncomfortable positions, cold environment, humid environment, adequate rest, or regular rest breaks. However, a significant association was observed between high work pace and MSDs, with participants reporting a high pace of work being more likely to report MSDs (χ² = 6.10, p = 0.013).

**Table 2:** Relationship between participants characteristics and work-related factor with musculoskeletal disorders among nurses (N = 172) by chi-squared.

VARIABLE	CATEGORY	MSDs, YES	MSDs, NO	χ²	χ² P-VALUE
**Education**	Diploma	31	1	0.936	0.884*
	Bachelor	110	9		
	Master or above	20	1		
**Job title**	Practical nurse	30	1	0.770	0.919*
	Registered nurse	114	8		
	Supervisor	8	0		
	Head nurse	9	1		
**Gender**	Male	97	5	3.5	0.334
	Female	64	6		
**Marital status**	Married	127	5	6.4	0.011
	Unmarried	34	6		
**BMI**	Underweight	5	0	4.7	0.134*
	Normal	103	10		
	Overweight	44	0		
	Obese	9	1		
**Work-related factors**					
**Overtime**	No	22	2	0.770	0.676
	Yes	139	9		
**Workspace**	No	109	6	0.175	0.370
	Yes	52	5		
**Lumbar support**	No	50	2	0.809	0.368
	Yes	111	9		
**Adjustable bench**	No	61	3	0.496	0.481
	Yes	100	8		
**Posture freedom**	No	85	5	0.012	0.910
	Yes	76	6		
**Same posture**	No	100	8	0.496	0.481
	Yes	61	3		
**Uncomfortable posture**	No	41	4	0.633	0.426
	Yes	120	7		
**Cold environment**	No	102	8	0.392	0.531
	Yes	59	3		
**Humid environment**	No	52	6	2.2	0.131
	Yes	109	5		
**Enough rest**	No	40	5	2.26	0.132
	Yes	121	6		
**Regular rest breaks**	No	53	4	0.055	0.814
	Yes	108	7		
**Stress**	No	32	4	1.6	0.193
	Yes	129	7		
**High work pace**	No	35	6	6.1	0.013*
	Yes	126	5		

*Fisher exact was used for Marital, BMI, and Job title because some cells have small expected counts.

For continuous variables, independent t-tests in Table 3 indicated no statistically significant differences in continuous variables between nurses with and without MSDs. Nurses with MSDs had a mean age of 33.1 ± 6.2 years compared to 32.0 ± 5.9 years among those without MSDs (p = 0.688). Similarly, years of work experience (9.3 ± 5.5 vs. 7.8 ± 6.5; p = 0.787), **number of displacement episodes (7.0 ± 4.6 vs. 5.7 ± 4.3; p = 0.813)**, and average daily working hours (11.2 ± 5.4 vs. 10.7 ± 3.9; p = 0.616) did not differ significantly between the two groups. Although nurses reporting MSDs tended to be slightly older, had longer work experience, higher displacement frequency, and longer daily working hours, these differences did not reach statistical significance.

**Table 3:** Relationship between participants characteristics and work-related factor with musculoskeletal disorders among nurses (N = 172) by t-test.

CONTINUOUS VARIABLE	MEAN ± SD (MSDs, YES)	MEAN ± SD (MSDs, NO)	T-TEST	P-VALUE
**Age (years)**	33.1 ± 6.2	32 ± 5.9	−0.489	0.688
**Experience (years)**	9.3 ± 5.5	7.8 ± 6.5	−0.797	0.787
**Displacement (episode)**	7 ± 4.6	5.7 ± 4.3	−0.891	0.813
**Working hours per day**	11.2 ± 5.4	10.7 ±3.9	−0.295	0.616

Overall, these findings suggest that MSD occurrence in this cohort may not be primarily driven by demographic characteristics or cumulative work exposure alone, but rather by other occupational, ergonomic, and psychosocial factors inherent to the working conditions during the prolonged conflict. Ergonomic factors, specifically the pace of work, may play a more important role in the development of MSDs among the participants. This emphasizes the need for workplace interventions targeting workflow management and ergonomic optimization to reduce MSD risk.

### Regression analysis of factors associated with musculoskeletal disorders

#### Univariable analysis

Univariable negative binomial regression was used to examine crude associations between each potential predictor and the number of MSD-affected body regions reported over the preceding 12 months. This approach was selected to maintain consistency with the primary multivariable model and to account for overdispersion in the count outcome.

In univariable analyses (Table 4), marital status was associated with a lower MSD count (Incidence Rate Ratio (IRR) = 0.73, 95% CI: 0.59–0.92, p = 0.007), indicating that married nurses have 29% fewer affected body regions compared with unmarried nurses, holding other variables constant. Conversely, exposure to a humid work environment was associated with a higher MSD count (IRR = 1.26, 95% CI: 1.04–1.54, p = 0.018) means the count of affected body regions increases by 26% compared with the reference group. BMI category, insufficient rest, and hard work pace showed weak or borderline associations, whereas perceived work-related stress was not associated with MSD count.

**Table 4:** Predictors of musculoskeletal disorders count (number of affected body regions).

VARIABLE	UNIVARIABLE IRR (95% CI)	P-VALUE	MULTIVARIABLE aIRR (95% CI)	P-VALUE
Marital status (married vs. not married)	0.73 (0.59 –0.92)	0.007	0.71 (0.57–0.88)	0.002
BMI category	0.88 (0.76 –1.02)	0.089	0.87 (0.76–1.00)	0.058
Adequate rest (yes vs. no)	0.83 (0.67–1.02)	0.079	0.82 (0.67–0.99)	0.045
Humid work environment (yes vs. no)	1.26 (1.04–1.54)	0.018	1.24 (1.03–1.50)	0.026
Work-related stress (yes vs. no)	1.15 (0.91–1.44)	0.235	1.03 (0.81–1.30)	0.833
Hard work pace (yes vs. no)	1.22 (0.98–1.52)	0.076	1.06 (0.84–1.34)	0.646

aIRR: adjusted incidence rate ratios.

#### Multivariable analysis

Variables with p-values <0.20 in the univariable analyses, together with known confounders from the literature, were included in the multivariable negative binomial regression model. The model estimated adjusted incidence rate ratios (aIRR) controlling for potential confounding among predictors. In the adjusted model, marital status (aIRR = 0.71, 95% CI: 0.57–0.88, p = 0.002), adequate rest (aIRR = 0.82, 95% CI: 0.67–0.99, p = 0.045), and workplace humidity (aIRR = 1.24, 95% CI: 1.03–1.50, p = 0.026) remained independently associated with MSD count. BMI category showed a borderline association, while work-related stress and hard work pace were not independently associated. All findings were interpreted cautiously given the exploratory nature of the analyses and the study’s sample size.

### Multicollinearity assessment

Prior to multivariable regression analysis, a multiple linear regression model was performed including all potential predictors of MSDs to assess multicollinearity. VIF ranged from 1.05 to 3.10, with a mean VIF of 1.52, indicating low multicollinearity and confirming that all variables could be included in subsequent negative binomial regression models without concern for collinearity. All VIF values were below 5, suggesting acceptable collinearity among predictors. The highest VIFs were observed for years of experience (3.10) and age (3.09), which is expected due to natural correlation.

## Discussion

This cross-sectional study of 172 critical care nurses working in Gaza City hospitals during the 2023–2025 armed conflict demonstrates an exceptionally high burden of MSDs. The 12-month prevalence was highest in the neck (69.2%), lower back (68.0%), and shoulders (64.5%), with the greatest pain intensity reported for the lower back. While these anatomical patterns are consistent with global evidence indicating that nursing-related MSD prevalence often exceeds 50–75% [5–8], the magnitude and functional impact observed in this cohort must be interpreted within the context of prolonged conflict, disrupted healthcare delivery, and extreme workload demands.

The predominance of bilateral symptoms in the shoulders, knees, and ankles suggests generalized mechanical strain rather than localized overuse. In conflict-affected hospitals, this pattern likely reflects cumulative exposure to prolonged standing, repeated manual patient handling, sustained awkward postures, and limited opportunities for task rotation or recovery during extended shifts. Similar mechanisms have been described in non-conflict settings; however, in Gaza, these exposures are amplified by staff shortages, mass-casualty surges, and damaged infrastructure that limit ergonomic optimization [9, 10].

Among the examined occupational factors, having adequate rest emerged as the most consistent protective correlate of MSD burden. Nurses reporting adequate rest experienced fewer affected body regions, underscoring the importance of recovery opportunities in mitigating cumulative musculoskeletal strain.

High work pace showed a non-significant but directionally consistent association with MSD presence and severity across analytical models, aligning with existing evidence linking time pressure, high task density, and insufficient recovery to musculoskeletal strain [11, 12]. In conflict settings, work pace is not solely organizational but is driven by unpredictable patient influx, emergency redeployments, and prolonged crisis operations. Although these contextual pressures may plausibly amplify musculoskeletal risk, their effects should be interpreted cautiously in light of the exploratory nature of the analyses.

Environmental conditions—particularly perceived workplace humidity—were independently associated with higher MSD counts. While humidity is a recognized occupational stressor in some non-conflict regions, in Gaza this exposure may be compounded by conflict-related infrastructure damage, inadequate ventilation, intermittent electricity, and overcrowded wards. Such conditions may exacerbate fatigue, discomfort, and perceived exertion during manual tasks, indirectly increasing musculoskeletal symptom burden [13, 14]. This finding highlights the relevance of environmental determinants that are often overlooked in MSD research but may be especially salient in crisis-affected health facilities.

In our study, nearly 60% of participants were male, which diverges from the gender distribution typically seen in nursing workforces in many high-income settings where females predominate. This reflects the local workforce composition in Gaza, where nursing—including in critical care settings—has a higher proportion of male clinicians, a pattern that is shaped by regional sociocultural norms and the demands of working in an active conflict environment [15]. Although some non-conflict literature suggests gender differences in MSD prevalence, findings are inconsistent and often context-specific. In our cohort, gender was not significantly associated with MSD count in either univariable or multivariable models, indicating that occupational and environmental exposures common to all nurses—such as prolonged high work pace, awkward postures, and adverse environmental conditions—are likely the dominant drivers of MSD burden rather than gender alone.

Marital status was associated with MSD burden, with married nurses showing slightly lower counts of affected body regions in the multivariable model. This suggests that social and family support may provide some buffering against cumulative musculoskeletal strain in the context of conflict. These findings highlight the potential role of psychosocial and contextual factors in moderating occupational risk in crisis-affected healthcare settings. In conflict settings marked by displacement and insecurity, such support networks may be particularly protective, underscoring the importance of contextual and psychosocial modifiers of occupational risk [14–16].

Ergonomic conditions were uniformly poor, with widespread reports of sustained trunk and neck flexion, twisting, and limited postural variability. Although multifaceted ergonomic interventions have shown effectiveness in reducing MSDs in stable settings [17, 18], their implementation in conflict zones is constrained by resource scarcity and operational instability. Nonetheless, low-cost and adaptable measures—such as task rotation, micro-breaks, basic assistive devices, and staff education—remain relevant and warrant evaluation in humanitarian and crisis contexts [19].

## Conclusion

Nurses working in Gaza City hospitals during the 2023–2025 conflict experienced a very high burden of MSDs, particularly affecting the neck, lower back, and shoulders. Multivariable analysis identified humid work environments as a risk factor, whereas being married and having adequate rest were protective against cumulative MSD burden. Other factors, including work pace, stress, and BMI, were not independently associated. These findings underscore the urgent need for context-adapted occupational health interventions, including ergonomic optimization and rest facilitation, to preserve nurse wellbeing and maintain workforce capacity in conflict-affected healthcare systems.

### Strengths and limitations

Key strengths include the focus on frontline nurses working under active conflict conditions, use of validated instruments, and complementary regression approaches. Limitations include the cross-sectional design, reliance on self-reported data, and non-probability sampling necessitated by wartime constraints, which limit causal inference and generalizability. Some variables, including humidity and marital status, may act as proxies for unmeasured contextual or psychosocial factors, a challenge commonly noted in MSD research in resource-limited and crisis settings [16–20].

### Implications for practice and research

The findings highlight several practical and research priorities for conflict-affected hospital settings. From a practice perspective, interventions should focus on mitigating environmental risk factors, such as poor ventilation and high humidity, and facilitating adequate rest periods during shifts to reduce MSD burden. Promoting social and organizational support may further strengthen protective effects, particularly for married nurses. From a research perspective, future studies should explore context-specific, low-cost ergonomic and organizational strategies that are feasible in high-demand, resource-constrained environments. Additionally, qualitative research could clarify mechanisms through which rest, marital status, and environmental conditions influence musculoskeletal health among nurses working under extreme stress.

## Data Availability

The datasets generated and/or analyzed during the current study are available from the corresponding author on reasonable request.

## Additional File

The additional file for this article can be found as follows:
