# Supplementary material for: Occupational and Environmental Determinants of Musculoskeletal Disorders Among Nurses in Conflict-Affected Gaza Hospitals
Source: Ann Glob Health. 2026 Jan 20;92(1):6. doi: 10.5334/aogh.5055 (PMC12829455; doi:10.5334/aogh.5055)
Supplement: Supplementary File 1. — Tables. [file agh-92-1-5055-s1.pdf]

Supplementary File 1

Functional movements and postures among participants (N = 172)

Domain				
Trunk	Trunk posture at work	Flexion 0–20°	2	1.2
		Flexion 20–60°	47	27.3
		Flexion >60°	116	67.4
		Extension	7	4.1
	Keep bending for long time	Yes / No	133 / 39	77.3
	Turn round frequently	Yes / No	137 / 35	79.6
	Keep trunk twisted for long time	Yes / No	119 / 53	69.2
	Bend and turn simultaneously	Yes / No	124 / 48	72.1
Neck	Neck posture at work	Flexion <10°	27	15.7
		Flexion 10–20°	89	51.7
		Flexion >20°	56	32.6
		Extension	0	0
	Head remains low for long time	Yes / No	136 / 36	79.1
	Keep neck twisted for long time	Yes / No	124 / 48	72.1
	Turn head frequently	Yes / No	136 / 36	79.1
Arm	Flex / extend wrist frequently	Yes / No	142 / 30	82.6
	Twist arm frequently	Yes / No	110 / 62	64.0
	Use support device in forearm	Yes / No	67 / 105	38.9
	Keep wrist twisted long time	Yes / No	81 / 91	47.1 9
	Place arm on edges	Yes / No	93 / 79	54.1
	Keep shrugging long period	Yes / No	79 / 93	45.9
	Tool size suitable for hand	Yes / No	82 / 90	47.7
	Operate with both hands	Yes / No	108 / 64	62.8
	Arm height	Below shoulder / Above shoulder	104 / 68	60.5 / 39.5
Leg	Sitting posture	Yes / No	124 / 48	72.1
	Keep both legs upright	Yes / No	48 / 124	27.9
	Keep standing for long time	Yes / No	81 / 91	47.1
	Keep legs bent or twisted for long time	Yes / No	91 / 81	52.9

Work Environment Characteristics among Nurses (N = 172)

Variable	Category	n (%)
Often work overtime	Yes	148 (86.1)
Enough workspace	Yes	57 (33.1)
Lumbar support available	Yes	52 (30.2)
Adjustable workbench	Yes	64 (37.2)
Can change posture freely	Yes	91 (52.9)
Keeps same posture most of time	Yes	108 (62.8)
Feels posture uncomfortable	Yes	127 (73.8)
Feels cold at work	Yes	62 (36.1)
Feels humid at work	Yes	114 (66.3)
Has enough rest time	Yes	45 (26.2)
Can rest regularly	Yes	57 (33.1)
Feels stressed at work	Yes	136 (79.1)
Finds it hard to keep work pace	Yes	131 (76.2)

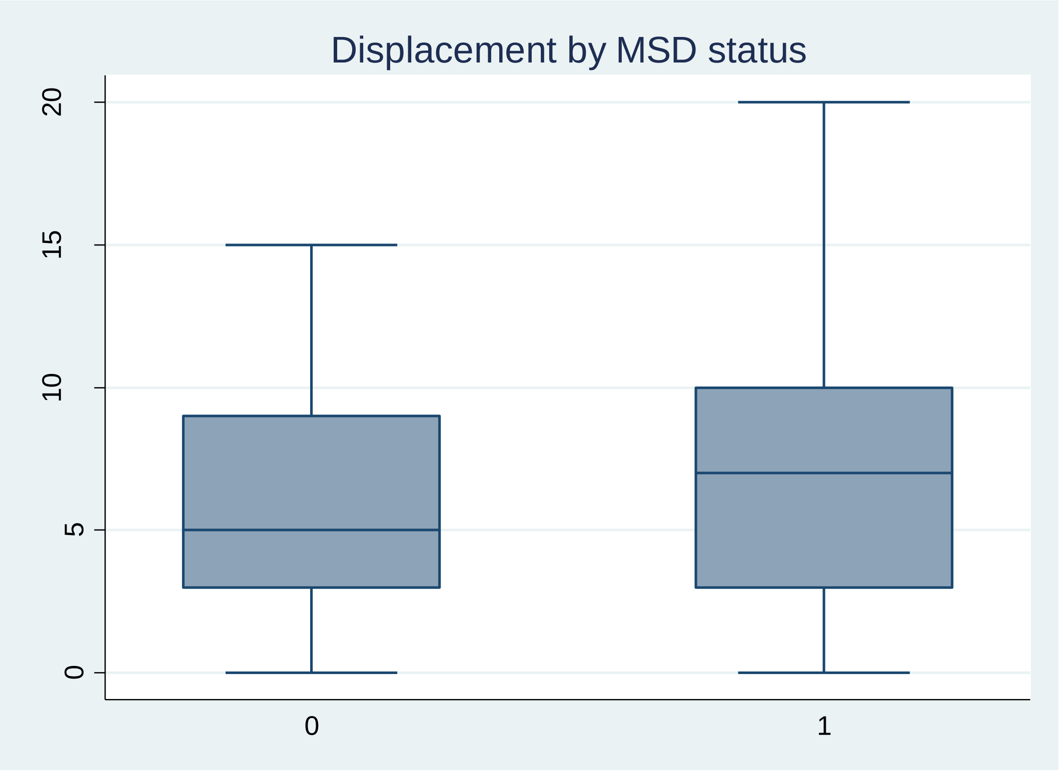

Figure 1: Distribution of displacement episodes among nurses with (1) and without (0) musculoskeletal disorders (t-test, p = 0.813).
